# Stress Hormones Cortisol and Aldosterone, and Selected Markers of Oxidative Stress in Response to Long-Term Supplementation with Omega-3 Fatty Acids in Adolescent Children with Depression

**DOI:** 10.3390/antiox11081546

**Published:** 2022-08-10

**Authors:** Henrieta Oravcova, Barbora Katrencikova, Iveta Garaiova, Zdenka Durackova, Jana Trebaticka, Daniela Jezova

**Affiliations:** 1 Institute of Experimental Endocrinology, Biomedical Research Center, Slovak Academy of Sciences, Dubravska Cesta 9, 84505 Bratislava, Slovakia; 2 Department of Pharmacology and Toxicology, Faculty of Pharmacy, Comenius University Bratislava, Odbojarov 10, 83104 Bratislava, Slovakia; 3 Institute of Medical Chemistry, Biochemistry and Clinical Biochemistry, Faculty of Medicine, Comenius University, Sasinkova 2, 81372 Bratislava, Slovakia; 4 Research and Development Department, Cultech Ltd., Unit 2 Christchurch Road, Port Talbot SA12 7BZ, UK; 5 Department of Paediatric Psychiatry, Faculty of Medicine, The National Institute of Children’s Diseases, Comenius University, Limbová 1, 83340 Bratislava, Slovakia

**Keywords:** mood disorders, hypothalamic-pituitary-adrenocortical axis, neuroendocrine function, diurnal variation, oxidative stress, omega-3 fatty acids

## Abstract

Late childhood and adolescence are crucial periods of brain development with high vulnerability to environmental insults. The aim of this study was to test the hypotheses that in adolescents with depression (a) 12 weeks-supplementation with omega-3 fatty acids results in the attenuation of salivary stress hormone concentrations; (b) the mentioned supplementation improves potentially disrupted daily rhythm of stress hormones; (c) stress hormone concentrations correlate with values of selected markers of oxidative stress. The sample consisted of 60 patients suffering from depression aged 11–18 years. Hormone concentrations in saliva were measured in the morning and midday before (baseline) and after (6, 12 weeks) food supplementation with omega-3 or omega-6 (as comparator) fatty acids. Morning cortisol decreased in response to omega-3 but not omega-6 fatty acids at 12 weeks compared to baseline. No changes were observed in aldosterone concentrations. The obtained results show that adolescent children with depression preserved the daily rhythm of both stress hormones. Baseline morning cortisol concentrations correlated positively with depression severity and lipoperoxides, and negatively with docosahexaenoic acid. Aldosterone concentrations correlated positively with 8-isoprostane. Thus, both hormones showed positive correlation with the selected markers of oxidative stress suggesting that enhanced stress hormone secretion may be associated with increased oxidative tissue damage in adolescent children with depression. This study was registered with the ISRCTN registry (DEPOXIN study, ISRCTN81655012).

## 1. Introduction

Despite intensive research, the exact pathophysiology and thus optimal treatment options for depressive disorders are still largely unknown. Among the pathophysiological mechanisms which are intensively investigated, there are two physiological processes referred to by the word stress. At the whole-body level, “stress” is the neuroendocrine response to aversive stressful stimuli, which aims to overcome demanding situations [1]. The stress response has hormonal, neural, immune, metabolic, mental, and behavioral components [2]. Inadequate coping with chronic stress situations is a risk factor for the development of a number of stress-related disease states, including major depression [3,4]. At the tissue level, the overproduction of free radicals and lipid peroxidation leads to “oxidative stress” representing oxidative tissue damage with negative consequences for health and disease including mental disorders [5,6,7].

A neuroendocrine correlate of the stress response considered to be involved in the pathophysiology of depression is the stress hormone cortisol. Cortisol is the executive component of the hypothalamic-pituitary-adrenocortical (HPA) axis [8,9]. With respect to affective disorders, both hypo- and hypercortisolism are associated with depression [10,11]. The potential changes in cortisol secretion during childhood and adolescence are even less understood. The first systematic review and meta-analysis looking at cortisol and major depressive disorder specifically in adolescents and young adults brought evidence suggesting that elevated morning cortisol concentrations were associated with an increased risk of developing adolescent depression [12]. With regard to other stress response components in children and adolescents, there are some studies on the activity of salivary alpha-amylase, a marker of the sympathetic nervous system [13,14], but no information appears to be available on the stress hormone aldosterone. Late childhood and adolescence are crucial periods of brain development [15] with high vulnerability to environmental insults. It is therefore important to conduct further research to better understand the biological mechanisms of stress and depression in adolescence and late childhood.

One of the approaches to enhancing the pharmacological treatment of depression is food supplementation with polyunsaturated fatty acids [16]. Food supplementation with omega-3, but not omega-6 fatty acids for twelve weeks was found to induce a significant reduction in the clinical signs of depression in late childhood and adolescence [17]. The improvement of the treatment outcome by omega-3 fatty acids supplementation in the mentioned group of adolescent patients was accompanied by a reduction in several oxidative stress markers [18]. The possible influence of omega-3 fatty acid supplementation on stress hormone concentrations is largely unknown.

The main objective of the present study was to test the hypothesis that long-term (12 weeks) food supplementation with omega-3 fatty acids in adolescent children with depression results in the attenuation of salivary stress hormone concentrations. The secondary hypothesis is that the mentioned food supplementation improves the potentially disrupted daily rhythm of stress hormones in youth with depression. An additional hypothesis is that changes in stress hormone concentrations correlate with changes in selected markers of oxidative stress. The results were evaluated in the whole sample of patients, as well as in stratified groups according to the diagnosis, namely either mixed anxiety and depressive disorder or depressive disorder.

## 2. Materials and Methods

The results of salivary hormone concentrations presented in this work are part of the DEPOXIN project (ISRCTN81655012). A detailed description of the study design, enrolment of patients, and their treatments as well as previous results were presented in recent publications [17,18,19,20].

### 2.1. Subjects

**Figure 1:**
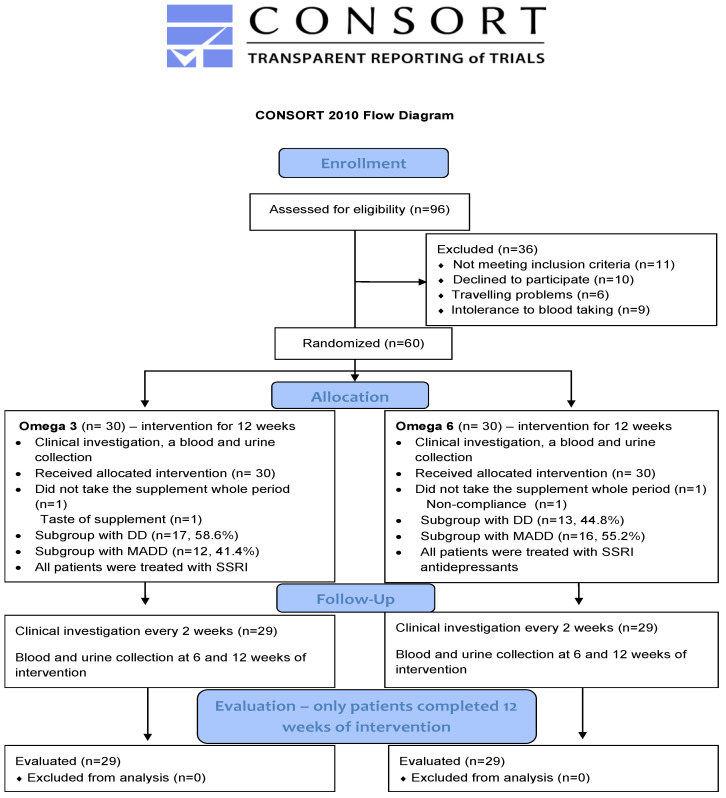
CONSORT 2010 Flow diagram. DD—depressive disorder; MADD-mixed anxiety and depressive disorder.

Briefly, a total of 80 boys and girls between the age of 11–18 years (patients and healthy controls) participated in this prospective study. The study group was compiled of 60 patients suffering from either mixed anxiety and depressive disorder (MADD) (*n* = 29) or depressive disorder (DD) (*n* = 31). All of them were registered at the Department of Paediatric Psychiatry of the Faculty of Medicine of Comenius University and The National Institute of Children’s Diseases in Bratislava and met the inclusion criteria which were an age of 11–18 years and diagnosis of MADD or DD according to the ICD-10 (International Classification of Diseases). The exclusion criteria involved chronic somatic diseases (autoimmune, endocrine, and metabolic), dietary restrictions (vegetarians, lactose intolerance), developmental disorders, and psychiatric comorbidity such as psychotic disorders, eating disorders, personality disorders, and addiction to psychoactive compounds. The 20 healthy children were included in the control group. All of them were registered in the Paediatric Centre Juvenalia. Dunajska Streda, Slovakia and were matched to patients with respect to their age. Healthy children were not subjected to any oil treatment and were sampled only at the baseline. The process of enrollment, allocation, follow up and evaluation is shown on the CONSORT flow diagram (Figure 1).

### 2.2. Interventional Study Design

Patients were randomly divided into two groups to receive either omega-3 fatty acids (referred to as OMG 3 group) or omega-6 fatty acids as an active comparator/placebo (referred to as OMG 6 control group) in addition to their treatment with selective serotonin reuptake inhibitors (SSRIs). As a source of omega- 3 fatty acids, a fish oil emulsion (Cultech Ltd., Port Talbot, UK, providing a daily dose of 2400 mg of total omega-3 fatty acids; 1000 mg eicosapentaenoic acid (EPA) and 750 mg docosahexaenoic acid (DHA), EPA:DHA ratio = 1.33:1) and omega-6, a sunflower oil (Cultech Ltd., Port Talbot, UK, providing a daily dose of 2467 mg of omega-6 linoleic acid in triacylglycerol form) were used. Patients in both groups received a daily dose of 20 mL of oil emulsion for 12 weeks.

### 2.3. Saliva Sampling and Hormone Analysis

Saliva samples were collected from each subject to determine hormone concentrations. Six saliva samples were collected from each patient. Sampling took place at three specific time intervals in the morning and at midday. The three specific time intervals were at the beginning of the study (week 0), in the middle of the intervention with fatty acids (week 6), and at the end of the intervention with fatty acids (week 12). Concerning the group of healthy children, saliva samples were taken only twice (morning and midday) at the baseline without any intervention.

The saliva samples of patients were collected in a children’s psychiatric outpatient clinic. The samples of healthy children were obtained in a pediatrician’s office during a regular visit. Morning sample collection was performed after overnight fasting between 8:00–8:30 am, at least 90 min after awakening. Children were asked to brush their teeth earlier than 60 min before saliva collection. Midday sample collection was performed at 11:00–11:30 a.m. on the same day as the morning sample. Children were instructed to avoid eating 60 min before saliva collection. Subjects were asked to stay in a sitting position for at least 20 min before and during sampling. They were asked to gently chew cotton swabs from Salivette sampling tubes (Salivette^®^ device, Sarstedt, UK) for 1 min. The samples were stored at −20 °C until analyzed.

Cortisol concentrations in saliva were determined using a commercially available enzyme-linked immunosorbent assay (IBL International, Hamburg, Germany). The intra- and inter-assay coefficients of variation were 2.9% and 5.0%, respectively.

Salivary aldosterone concentrations were measured by a modified methodology using a commercial radioimmunoassay kit (Immunotech, Prague, Czech Republic). The intra- and inter-assay coefficients of variation were 9.5% and 9.9%, respectively.

### 2.4. Oxidative Stress Markers

Oxidative stress markers, in other words, markers of oxidative tissue damage, determined in children enrolled in the DEPOXIN study were recently published [18] with details of the methods used. Similarly, the severity of depressive symptoms (expressed as the Children’s Depression Inventory, CDI score) and ratios of serum omega-6/omega-3 fatty acids as well as effects of fatty acids on the mentioned parameters have already been published [17].

### 2.5. Statistical Analyses

Differences between patient values and healthy controls were analyzed by performing an unpaired t-test or a non-parametric, Mann–Whitney U test depending on data normality. The morning and midday hormone values were compared using the Wilcoxon matched-pairs signed-ranks test. The effect of omega-3 fatty acids and omega-6 fatty acids was determined by performing the Friedman test. The subsequent Conover pairwise comparison test was used to compare data at weeks 6 and 12 of the intervention against the baseline. The correlation analyses were performed using Spearman’s rank test.

We used StatsDirect^®^ 3.3.4 (StatsDirect, Ltd., Birkenhead, Mersey-side CH428NQ, UK) for statistical analysis. The results are expressed as dot plots with each dot representing an individual subject with the mean ± SEM. The overall level of statistical significance was defined as *p* < 0.05.

## 3. Results

### 3.1. Baseline Data

Baseline characteristics of patients enrolled in the DEPOXIN project and healthy controls such as age, weight, height, and BMI (kg/m^2^) are provided in our previous publications [17,18,19,20].

### 3.2. Comparison of Salivary Hormone Concentrations in Patients and Healthy Subjects

**Figure 2:**
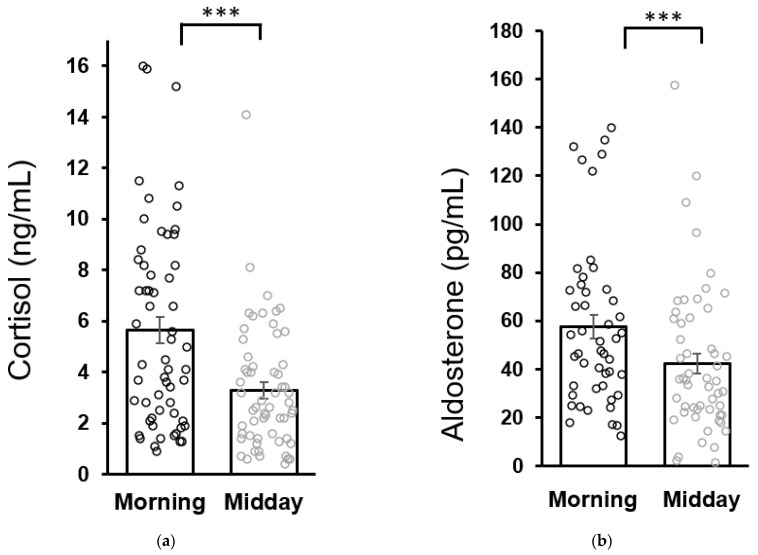
Circadian variation of salivary cortisol (**a**) and aldosterone (**b**) secretion in patients at the baseline (week 0). Statistical significance as revealed by Wilcoxon test: *** *p* < 0.001. Results are expressed as dot plots with each dot representing an individual subject with means ± SEM represented by horizontal lines.

No differences were found in baseline morning cortisol concentrations between patients (5.6 ± 0.5 ng/mL) and healthy children (6.0 ± 0.7 ng/mL). Similarly, baseline morning aldosterone concentrations in patients (57.7 ± 4.8 pg/mL) were not different from the healthy controls (43.3 ± 6.7 pg/mL as analyzed by the Mann–Whitney U test). Both cortisol and aldosterone concentrations in patients were lower at midday compared to the morning values which is consistent with the circadian variation of cortisol and aldosterone secretion (Figure 2).

### 3.3. Effect of Fatty Acids Supplementation in Patients with Depression

The effect of omega-3 and omega-6 FA on the severity of depression has already been investigated [17]. Briefly, all pairwise comparisons according to Conover in the Friedman test in the OMG 3 group showed highly significant differences in CDI in the study weeks from the baseline (from *p* = 0.001 in the 2nd week to *p* < 0.0001 at week 12). The highest reduction in the CDI score in the OMG 3 group was observed after 10 weeks of intervention (−7.6 = −27.4% of baseline score).

In the OMG 6 group, no significant effects of either the time-dependent treatment determined by the Friedman test (*p* = 0.372) or the Conover pairwise comparison (*p* > 0.05) were observed. The highest reduction in the CDI score in the OMG 6 group was observed after 12 weeks of intervention (−2.9 = −11.8%).

**Figure 3:**
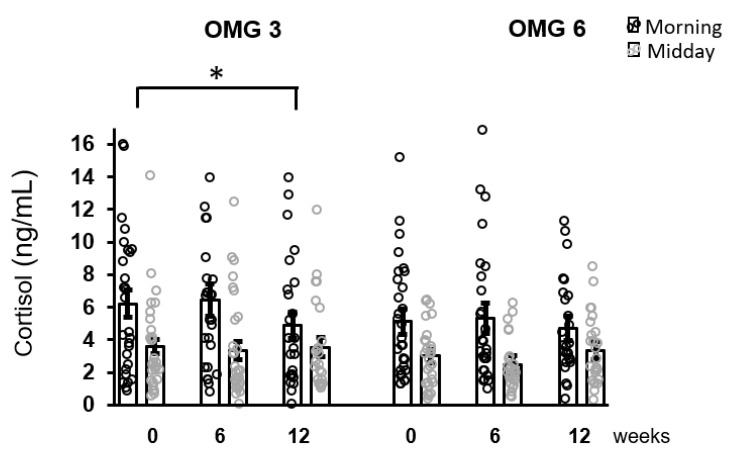
The effect of fatty acids supplementation on salivary cortisol concentrations in the whole sample of patients. A decrease in morning cortisol concentrations as revealed by Conover pairwise comparison test: * *p* < 0.05. Results are expressed as dot plots with each dot representing an individual subject with means ± SEM. OMG 3—omega-3 fatty acid, OMG 6—omega-6 fatty acid.

The effect of omega-3 fatty acids supplementation on morning cortisol concentrations over the intervention period (week 0, 6, 12) was only borderline significant (*p* = 0.055) with no change for midday cortisol levels (*p* = 0.168) as evaluated by performing the Friedman test. Using Conover pairwise comparison, a decrease in morning cortisol concentrations during the intervention period was observed at week 12 (*p* = 0.026) compared to the baseline (Figure 3).

**Figure 4:**
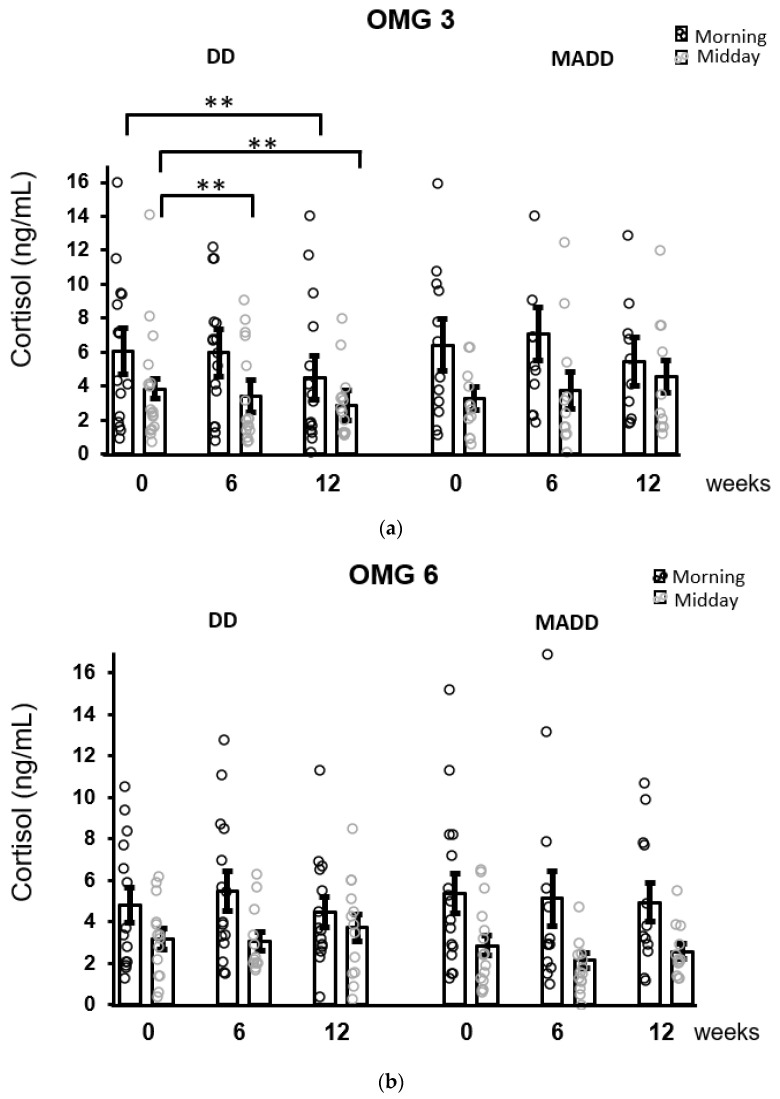
The effect of omega-3 (**a**) and omega-6 (**b**) fatty acids supplementation on salivary cortisol concentrations after stratification of patients according to the diagnoses. Decreases in morning and midday cortisol concentrations as revealed by Conover pairwise comparison test: ** *p* < 0.01. Results are expressed as dot plots with each dot representing an individual subject with means ± SEM. DD—depressive disorder subgroup and MADD—mixed anxiety and depressive disorder subgroup.

When the patients were stratified according to the diagnosis, the results of the Conover pairwise comparison showed that a decrease in morning salivary concentrations was evident in patients with DD (*p* = 0.004) and was not observed in patients with MADD following 12 weeks of intervention. Moreover, a decrease in cortisol concentrations over time in the DD subgroup was statistically significant (Conover test) in the midday samples at 6 (*p* = 0.008) and 12 (*p* = 0.001) weeks compared to baseline (Figure 4).

**Figure 5:**
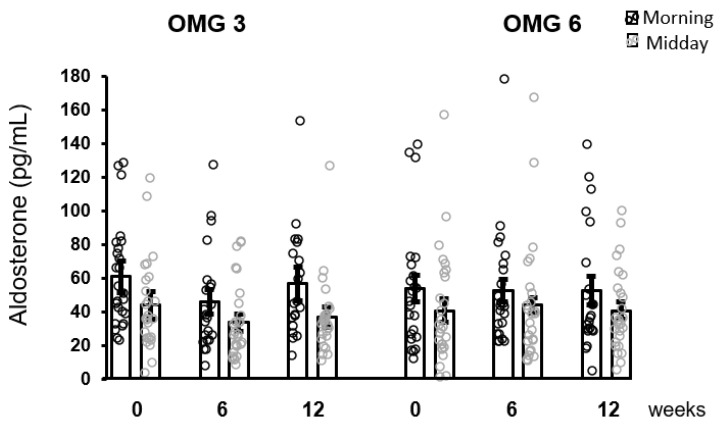
No effect of fatty acids supplementation on salivary aldosterone concentrations in the whole sample of patients. Results are expressed as dot plots with each dot representing an individual subject with means ± SEM. OMG 3—omega-3 fatty acid, OMG 6—omega-6 fatty acid.

As shown in Figure 5, the effect of omega-3 fatty acids as well as of omega-6 fatty acids supplementation in patients on aldosterone concentrations over the intervention time as analyzed by performing the Friedman test was not statistically significant at 6 and 12 weeks (*p* = 0.176 and *p* = 0.377, respectively).

### 3.4. Correlations between Parameters

The correlations between cortisol or aldosterone concentrations and selected parameters possibly related to neuroendocrine functions in children with depression were performed. These selected parameters were previously published, namely, the depression severity characterized by the CDI score [17], EPA and DHA levels, omega-6/omega-3 fatty acids ratio [17], thromboxane B, homocysteine and brain derived neutrophic factor (BDNF) [20] and oxidative tissue damage markers (lipoperoxides (LP), 8-isoprostane (8-IsoP), nitrotyrosine (NT), advanced oxidation protein products (AOPP), trolox equivalent antioxidant capacity (TEAC), Cu/Zn superoxide dismutase (SOD), glutathione peroxidase (GPx) and catalase (CAT)) [18].

At the baseline (week 0), there was a positive correlation between morning cortisol concentrations and CDI scores or LP values in patients (Table 1). In the same group, midday cortisol concentrations did not show any significant association with the selected parameters except a positive correlation with LP (r = 0.332, *p* = 0.007). Morning aldosterone concentrations in patients correlated positively only with 8-IsoP (Table 1).

In the control group, no significant correlations were found between morning cortisol and the omega-6/omega3 fatty acids ratio, 8-isoP, LP, NT and GPx activity at the baseline. Midday cortisol concentrations correlated positively (r = 0.396, *p* = 0.052) with NT values. Similarly, morning aldosterone in healthy controls did not correlate with the above-mentioned markers, except for a marginally significant negative correlation with NT (r = −0.347, *p* = 0.079). Midday aldosterone concentrations in healthy children positively correlated with NT values (r = 0.424, *p* = 0.034) but not with the other parameters investigated.

**Figure 6:**
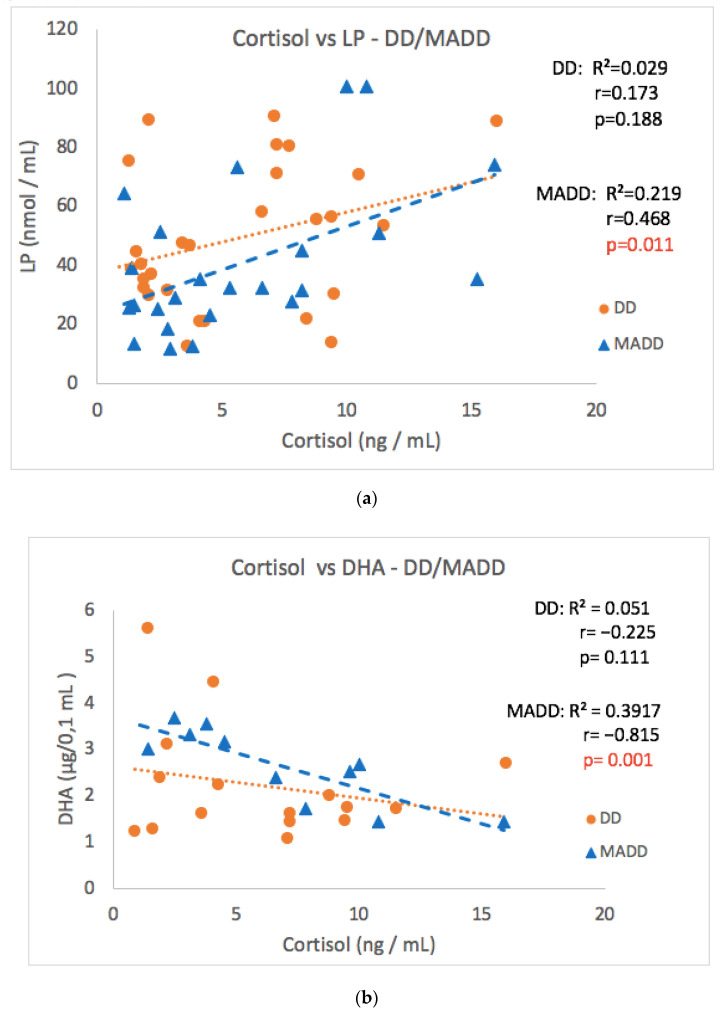
Correlations between morning cortisol concentrations versus LP (**a**) and DHA (**b**) at the baseline in the DD and MADD subgroups. LP–lipoperoxides, DHA–docosahexaenoic acid, DD–depressive disorder subgroup, and MADD–mixed anxiety and depressive disorder subgroup.

As the group of patients consisted of two diagnoses, the patients were divided according to the diagnosis to subgroups DD and MADD (Table 1). The significant correlation observed in the whole group of patients was not confirmed in subgroups DD or MADD between cortisol and the CDI score, 8-IsoP, NT, AOPP and SOD values (Table 1). However, a strong positive correlation between morning cortisol and LP values and a negative correlation between cortisol and DHA levels in the MADD subgroup was noticed (Figure 6a,b). Surprisingly, a negative correlation between morning cortisol and NT (Table 1) was found in the group of patients with MADD, but not with DD. A significant positive correlation of salivary aldosterone concentrations with 8-IsoP was observed only in patients with MADD.

## 4. Discussion

The obtained results confirmed the main hypothesis that long-term (12 weeks) food supplementation with omega-3 fatty acids in adolescents led to the attenuation of morning salivary cortisol concentrations. Following the stratification of patients according to the diagnosis, this decrease was not observed in patients with MADD but it was particularly strong in patients with DD. This is consistent with the conclusions of Trebaticka et al. [17], who found a more significant effect of omega-3 fatty acid in the DD subgroup compared to the MADD subgroup. In this subgroup of patients, a reduction in cortisol concentrations was found following six weeks of treatment and the effects were also statistically significant for midday hormone concentrations. The supplementation with omega-3 fatty acids failed to have an impact on the concentrations of salivary aldosterone. The adolescent children with depression showed an undisturbed diurnal pattern of both cortisol and aldosterone secretion. At the baseline, a significant positive correlation between morning cortisol concentrations and CDI scores in the whole cohort of patients was observed. Morning salivary aldosterone did not correlate with CDI scores in any group of patients, but it positively correlated with 8-IsoP. The highest number of associations between stress hormone cortisol concentrations and markers of oxidative stress was observed in patients with MADD. Cortisol correlated positively with LP and negatively with DHA, thus confirming the third hypothesis.

Long-term food supplementation with omega-3 fatty acids in the present cohort of adolescent children led to a reduction in morning salivary cortisol concentrations. These results are consistent with existing knowledge on the relationship between HPA-axis activity and fatty acid metabolism in recurrent depressive disorder in adults [21]. Adults with low omega-3 fatty acid levels showed a dysregulated HPA axis suggesting that the treatment with these fatty acids might improve somatic and mental health [22]. Indeed, dietary supplementation with polyunsaturated fatty acids reduced corticosterone concentrations in rats [23], and cortisol secretion in healthy subjects [24], as well as in adult patients with depression [21,25]. Twelve-week exposure to polyunsaturated fatty acids in the form of fish oil in healthy prepubertal children failed to modify salivary and hair cortisol concentrations [26]. The present finding of decreased salivary cortisol concentrations appears to be the first piece of evidence observed in children.

The decrease in salivary cortisol concentrations was clearly evident in adolescent children with DD, while it was absent in the subgroup with diagnosed MADD. The mechanisms responsible for this difference are unknown. In healthy prepubertal children, the presence of high-trait anxiety was found to modify cortisol responses to real-life stressors [27]. It may be suggested that the influence of increased anxiety in children with MADD on HPA axis activity overcrowded the action of omega-3 fatty acid supplementation.

The present results did not reveal differences in the stress hormone concentrations between patients and healthy children at baseline before the intervention. However, the obtained results show that adolescent children with depression have a preserved daily rhythm of both stress hormones, which was shown to be disturbed in adult patients with depression [28,29]. The lack of differences in cortisol concentrations between patients and healthy controls is surprising as a systematic review and meta-analysis reported elevated morning cortisol concentrations in adolescents with depression [12]. However, the comparison of baseline concentrations between patients and healthy children was not among the primary aims of the present study and the number of healthy adolescent children was low.

Based on our previous results obtained in adult patients with depression showing higher aldosterone concentrations compared to healthy subjects [11], we expected increased aldosterone concentrations in children. This was not the case. Further studies are needed to demonstrate a lack of increased aldosterone concentrations in children, as the number of healthy children in the present study was low. It is clear that supplementation with omega-3 fatty acids failed to modify concentrations of aldosterone in the saliva of adolescent children with depression, which to be best of our knowledge has not been reported yet.

Morning salivary cortisol concentrations at baseline before the intervention positively correlated with CDI scores in the whole cohort. This finding is generally consistent with the observation of Van den Berg and Calster [30] in postpubertal adolescents scoring high on the CDI. However, these authors reported the association between the degree of depressive symptomatology and the diurnal cortisol profile including evening cortisol values.

The results of the present study further show that there is no association between CDI score and salivary aldosterone. This is on the contrary to adult patients with depressive disorder, where morning salivary aldosterone concentrations reflected the severity of depression [28].

Adult patients with clinical depression exhibit increased markers of oxidative stress [31]. The results obtained for the same cohort of children with depression as included in the present study showed increased levels of selective markers of oxidative tissue damage [18] compared to those in healthy controls. An important finding of the present study is the positive correlation between baseline morning cortisol concentrations and the levels of lipoperoxides, early stage lipid peroxidation markers, observed in the whole cohort as well as in the sub-group of patients with MADD. Though both increased markers of oxidative stress and dysregulation of the HPA axis occur in depressive disorders [32], the relationship between these two systems has not been reported yet. This relationship is further supported by the original finding of the present study of a negative correlation between baseline morning cortisol concentrations and the DHA, which contributes to endogenous antioxidant status, which was observed in the subgroup of adolescent children with MADD. Somehow surprising is the negative correlation between cortisol and nitrotyrosine, a marker of protein damage in the MADD group, but not in the DD subgroup of patients, which was expected to be positive and therefore needs further verification.

Baseline morning aldosterone concentrations correlated positively with 8-isoprostane, a late-stage marker of lipid peroxidation, in both the whole cohort of adolescent children with depression as well as in the subgroup of patients with MADD. Thus, similarly to the cortisol, high aldosterone secretion might be associated with increased oxidative tissue damage in adolescent children with depression.

## 5. Conclusions

The present finding of decreased salivary cortisol concentrations appears to be the first piece of evidence on the effect of long-term food supplementation with omega-3 fatty acids observed in children with depression. Our results showed that adolescent children with depression have a preserved daily rhythm of both stress hormones, which appears to be disturbed in adult patients with depression. Supplementation with omega-3 fatty acids failed to modify concentrations of aldosterone in the saliva of adolescent children with depression, which has not been reported yet.

Both cortisol and aldosterone concentrations showed a positive correlation with selected markers of oxidative stress. It may be suggested that enhanced stress hormone secretion may be associated with increased oxidative tissue damage in adolescent children with depression.

## Figures and Tables

**Table 1:** Correlations between morning cortisol or aldosterone and parameters in the patients at the baseline (week 0).

Cortisol and	*n*	*r*	*p*	Aldosteron and	*n*	*r*	*p*
CDI	57	0.301	**0.015**	CDI	48	−0.091	0.269
LP	52	0.295	**0.017**	LP	43	0.055	0.363
8-IsoP	56	0.179	*0.093*	8-IsoP	47	0.342	**0.019**
NT	55	−0.198	*0.074*	NT	45	−0.06	0.692
AOPP	59	0.027	*0.055*	AOPP	48	0.022	0.441
TEAC	59	0.004	0.486	TEAC	49	−0.027	0.425
SOD	58	−0.182	*0.086*	SOD	49	0.135	0.177
GPx	59	0.101	0.222	GPx	49	−0.118	0.416
CAT	59	−0.061	0.323	CAT	48	0.012	0.935
TXB	55	−0.109	0.215	TXB	44	−0.057	0.711
HCy	58	−0.007	0.479	HCy	47	−0.001	0.497
BDNF	52	−0.067	0.319	BDNF	41	0.182	0.253
DHA	55	−0.197	*0.075*	DHA	47	0.163	0.137
EPA	55	−0.164	0.115	EPA	47	0.24	*0.052*
Patients accordig to diagnosis							
**Cortisol and**	*n*	*r*	*p*	**Aldosteron and**	*n*	*r*	*p*
CDI-DD	33	0.265	*0.068*	CDI-DD	25	−0.091	0.269
CDI-MADD	24	0.33	*0.057*	CDI-MADD	23	−0.175	0.212
LP-DD	28	0.173	0.188	8-IsoP-DD	25	0.222	0.284
LP-MADD	24	0.468	**0.011**	8-IsoP-MADD	22	0.516	**0.015**
NT-DD	32	0.101	0.291	GPx-DD	25	−0.006	0.978
NT-MADD	23	−0.591	**0.002**	GPx-MADD	23	−0.499	0.016
DHA-DD	31	−0.225	0.111				
DHA-MADD	23	−0.815	**0.001**				

CDI—Children’s Depressive Inventory, LP—lipoperoxid, 8-IsoP—8-isoprostane in urine, NT—nitrotyrosine, AOPP—advanced oxidation protein products, TEAC—trolox equivalent antioxidant capacity, SOD—superoxide dismutase, GPx—glutathione peroxidase, CAT—catalase, TXB—thromboxane B, HCy—homocysteine, BDNF -brain derived neutrophic factor, DHA—docosahexaenoic acid, EPA—eicosapentoenoic acide, DD—depressive disorder, MADD—mixed anxiety and depressive disorder, *n*—number of evaluated children, *r*—Spearman’s rank correlation coefficient, *p*—significance, data in bold are significant and in italics are marginally significant.

## Data Availability

Data is contained within the article.
